# Comparative Mechanical Study of Pressure Sensitive Adhesives over Aluminium Substrates for Industrial Applications

**DOI:** 10.3390/polym14214783

**Published:** 2022-11-07

**Authors:** Marta Ortega-Iguña, Mariane Chludzinski, José María Sánchez-Amaya

**Affiliations:** Department of Materials Science and Metallurgical Engineering and Inorganic Chemistry, School of Engineering, University of Cádiz, Av. la Universidad de Cádiz, 10, E-11519 Puerto Real, Cadiz, Spain

**Keywords:** pressure-sensitive adhesive, aluminium, surface roughness, surface chemical treatments, mechanical properties, creep testing

## Abstract

The use of adhesives for fixing low-weight elements is showing increasing interest in the industry, as it would reduce the weight of the assembly, costs, and production time. Specifically, the application of pressure-sensitive adhesives (PSAs) to join non-structural naval components to aluminium substrates has not yet been reported. In the present work, a study of the mechanical behaviour of different double-sided PSAs applied on bare aluminium alloy substrates is performed. The influence of surface roughness, surface chemical treatments, and the matrix of the adhesives is studied through different mechanical tests, such as shear, T-peel, and creep. The application of an adhesion promoter improved the mechanical behaviour. Low roughness substrates provided better performance than ground samples. Acrylic foam adhesives were subjected to creep tests, whose results were fitted to a simple mathematical model, predicting the fracture time as a function of the applied load.

## 1. Introduction

The industry currently faces different challenges, such as demands for higher performance, lower energy consumption, and the consolidation of more environmentally friendly processes. In this context, one strategy to reduce the emission of pollutants to the ecosystem is the use of lighter and more efficient materials [1,2,3]. This need justifies the rise in the use of aluminium alloys as a substitute for steel, due to their advantageous properties, among them high resistance, low density, resistance to corrosion, resistance to fatigue, and reduction in costs associated with the weight and fuel savings in transport industries [4,5]. However, the reduction in emissions is not limited to the use of lighter materials, but also seeks the use of lighter, simpler, and cheaper joining technologies. One of the most promising alternatives to welding techniques for some specific applications is adhesive bonding. This methodology is advantageous for manufacturing in an energy efficient way since it reduces the weight of the joints and allows the joining of a higher number of surfaces compared to traditional joining methods [6]. Therefore, there has been an increase in the demand for adhesives as an alternative material to traditional bonding processes [1,7,8,9]. Indeed, the adhesive bonding of aluminium is already widely used in different industries, such as automotive and aeronautics, because of their high performance, low cost, easy application, good damping behaviour, and resistance to corrosion [10,11,12]. There are important potential applications of adhesive joining in other industries, such as naval and offshore, in which the use of cold joining processes and light materials are highly demanded.

At present, the adoption of pressure sensitive adhesives (PSAs) is being frequently used because of their advantages, such as easy and safe application [13]. There are diverse types of PSAs that differ in the adhesive base, support material, as well as in their applications. These adhesives consist of flexible support with a permanent tack adhesive layer that adheres to most substrates under pressure. In addition, they can be easily removed without leaving residues on the surface [14]. Other important aspects are that the PSA does not change its physical state or require the application of solvents or heating before usage [10].

PSAs can be broadly classified into five categories: polyurethane, silicone, thermoplastic elastomer, rubber, and acrylic materials. It is important to note that the resistance of this type of adhesives not only depends on their nature but also on the degree of crosslinking of the polymer chains [10]. Non-crosslinked polymers are also widely used as PSAs, the resistance of which is improved by the introduction of fillers whose particles form a three-dimensional network [15,16]. Likewise, the use of nanotubes as filler particles in the adhesive matrix provides this three-dimensional network, which improves the mechanical properties of PSAs [15,16]. Among the groups of PSAs mentioned above, the most employed in the industrial sector are acrylic-based materials, with numerous families differing in the polymerization process (emulsion, solution, hot melt, or radiation curing) [17,18,19]. Within the wide range of available PSAs, recent developments have led to a new category, the so-called “high performance”. This range of PSAs combines a great capacity for cohesion, resistance, and moldability, even on rough or smooth surfaces, making them stand out from conventional PSA. To achieve maximum strength, these joints require adequate external applied pressure to cure properly, as well as the elimination of air bubbles that may be trapped in the PSA–substrate interface [10]. This type of adhesive also has the benefit of being able to assemble a large number of surfaces of diverse nature, such as polypropylene, stainless steel, glass, carbon-fibre reinforced polymer (CFRP), as well as aluminium [20,21,22,23,24]. In a recent study, a high-performance PSA was employed to join samples painted with a naval epoxy painting scheme [25]. The influence of the surface preparation, curing time, compression force, and compression time on the shear and tensile mechanical behaviour was investigated.

The adhesive characteristics are largely determined by studies of tack and mechanical properties. The most frequent mechanical properties evaluated in research studies are shear, tensile, and peeling [8,26,27]. The creep behaviour is also of great importance in the industry since this testing allows determining the optimal range of loads to be supported by the joints for a prediction of service life [28]. Previous creep studies of PSAs have been reported in [29,30,31]. The creep response is described to depend on the applied load and working temperature; the lifetime decreases as theses variables increase. Thus, Townsend et al. [29,30] studied the effect of humidity and temperature variables on the creep properties of acrylic foam PSAs. For their part, Yamaguchi et al. [31] developed a model to predict the creep behaviour of PSAs, taking into account factors such as viscoelasticity and cavitation dynamics of these adhesives.

Some studies investigate the fracture characteristics of PSAs, in terms of adhesion and cohesion, as a function of the energy dissipated by the polymer [32,33,34,35,36,37,38]. Achieving maximum mechanical resistance to these stresses requires the optimization of factors such as energy and surface roughness. The surface energy can be adjusted by applying excited gas discharge (corona), plasma, or chemical-based surface treatments [39]. These treatments introduce functional groups at the substrate–PSA interface, in addition to increasing wettability, thus promoting better adhesion [40,41]. However, despite the aforementioned, in order to maintain the behaviour of PSA at a high performance, it is necessary to keep the joined surfaces clean of impurities [42]. On the other hand, a medium–high roughness of the substrate can cause an incomplete joint, reducing the mechanical properties. This is related to the amount of air bubbles that can be trapped at the substrate–PSA interface, since these bubbles can cause cavities in the early stages of bond failure [43,44]. As a result, the type of failure is highly dependent on the presence of defects at the PSA–substrate interface. Moreover, the load applied to the joint also determines the failure pattern. 

Due to the demands of some industries for weight reduction, as naval and offshore sectors, the use of adhesives has increased. Some needs of these industries include cold, light, cheap, and fast joining technologies to bond not structural components, such as air ducts, silent blocks (resilience blocks to dampen vibrations), ventilation grills, and electrical cable supports. Most of the research carried out on aluminium substrates is limited to the use of structural adhesives, especially those of the epoxy and polyurethane type [2,26]. Furthermore, PSA adhesive bonding on aluminium substrates has not been extensively tested. The studies carried out are mainly focused on the optimization of the joints by means of different surface preparations applying different cleaning agents and chemical treatments before assembling. Likewise, in these studies, the response of the PSA aluminium joints was evaluated by modifying some parameters that intervene in the curing process, such as temperature. The efficiency of these treatments is interpreted through the response of the joints when they are subjected to shear stress [23,24]. The creep behaviour at different temperatures of one high-performance acrylic foam PSA applied to aluminium substrates for glazing applications is also investigated in detail in [29,30].

Considering the overall literature reviewed, it can be stated that there is a clear need for knowledge regarding the potential use of PSAs in industry. Concisely, their application to join non-structural components to aluminium substrates is not widely addressed, especially in shipbuilding applications. In order to cover this gap of knowledge, the present work investigates the response of PSA adhesives of different natures when they are applied on aluminium substrates with different roughness and surface chemical treatments. The mechanical properties of the joints are experimentally analysed by means of shear, T-peel, and creep tests.

## 2. Materials and Methods

AA5083-H111 and AA5754-H111 aluminium alloys, whose compositions are detailed in Table 1, were employed for this investigation. AA5083-H111 aluminium specimens (10 mm × 100 mm × 2 mm) were used to perform shear and creep tests. On the other hand, AA5754-H111 aluminium specimens (25 mm × 200 mm × 0.5 mm) were used for the T-peel test. The specimens of each test were defined according to ISO 13445, ISO 14678, and ISO 11339 standards, respectively. Silicon carbide sandpaper with different grain sizes (P40, P80, P320, P600, and P1200) was used to prepare the surface with different roughness, in which the sandpaper code corresponds to the number of abrasive particles per square inch. The arithmetic mean roughness (*R_a_*) was measured at least 5 times using an adapted roughness tester with a surface probe (Perthometer PGK 120, Mahr, Göttingen, Germany). The bonding area of all samples was cleaned with isopropanol before the assembling process. Furthermore, the influence of the use of a thin layer of an adhesion promoter based on a ciclohexane/xylene solution (Fast Cure Promoter, FCP 60153, TESA, Norderstedt, Germany) was also evaluated after the cleaning procedure.

**Figure 1:**
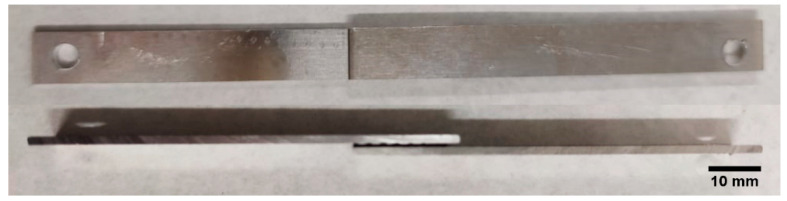
Aluminium joint assembled with the pressure-sensitive adhesive.

Single-lap joints (SLJ) were assembled with a rectangular area of 10 mm × 20 mm (Figure 1) using three different types of PSAs: a rigid acrylic foam (RF), a flexible acrylic foam (FF), and a flexible acrylic core (FC). Table 2 shows the thickness and the commercial name of each PSA. It also includes the density values, measured experimentally. FC adhesive is known to contain trimethylolpropane triacrylate (TMPTA) with a high degree of crosslinking. Meanwhile, RF and FF present a high content of methyl methacrylate (MMA). 

**Figure 2:**
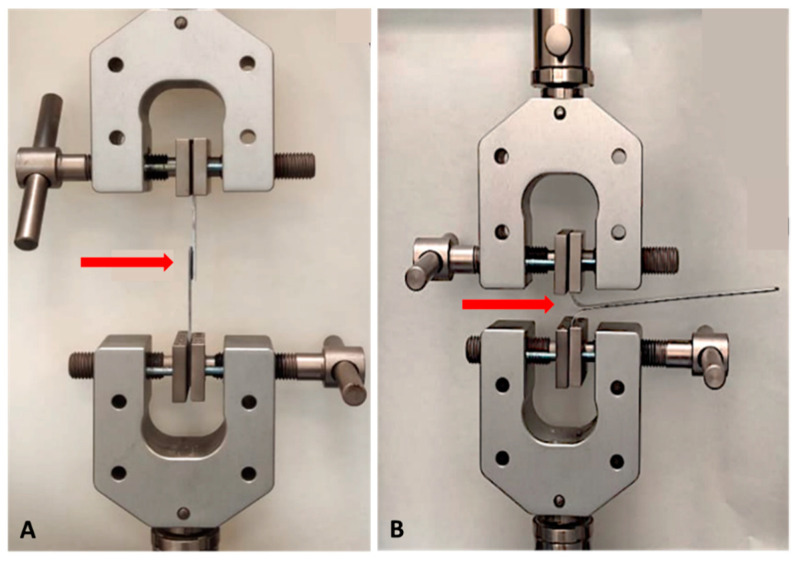
Example of adhesive shear (**A**) and T-peel (**B**) tests.

The joints were subjected to a compression force of 30 N/cm^2^ for 60 s, using a Shimadzu universal test equipment (10 kN of maximum load). Then, the joints were cured at room temperature (21 °C) and a controlled relative humidity of 60% for 72 h. After curing, the shear test was carried out at a constant speed of 10 mm/min, in accordance with ISO 13445. Figure 2A shows the experimental setup of the shear tests performed with the Shimadzu universal test equipment. The assembly of the joints was divided into two batches, with and without the application of adhesion promoter to the aluminium specimens prior to assembly. These shear tests allowed the study of the influence of the different surface roughness and chemical treatments on the mechanical performance of the three PSAs. In addition, the T-peel tests (Figure 2B) were performed in accordance with ISO 11339, employing the best surface conditions measured in the shear tests. All tests were carried out at least in triplicate to assure reproducibility. The failure mode was measured after testing according to ISO 10365, using image analysis software (Image-J, version 1.52q, National Institutes of Health, Bethesda, MD, USA). Ductility (elongation at fracture), and static toughness (overall area below the stress–strain curve, estimated by the 6th degree full polynomial that better fitted to each curve) were extracted from the stress–strain curves, obtained for each adhesive.

**Figure 3:**
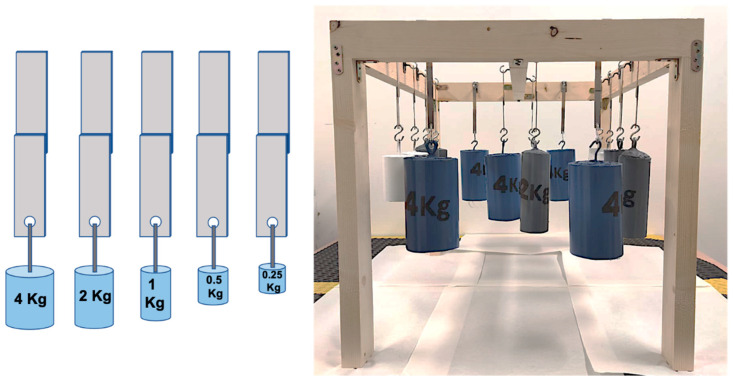
Creep tests illustration and setup.

The adhesive with the best properties in terms of shear and T-peel strength was selected to evaluate its creep resistance on specimens of the same dimensions as those used in the shear tests. The creep tests were performed at 23 °C and 70% of relative humidity, following the indications described in the ASTM D-1780 standard, allowing the measurement of the fracture time as a function of the applied load. The maximum load used in the creep testing was selected according to the peak strength obtained from the shear results (20 kg/cm^2^), with the other load values being a percentage of it. The applied load values are listed in Table 3 and Figure 3 illustrates the creep test setup.

## 3. Results and Discussion

### 3.1. Roughness

The surface roughness of the different sanded/ground aluminium surfaces was measured at least five times per condition, in different positions of the bonding area, to assure that measurements were representative of each ground level. The roughness values reported in the paper correspond to the mean arithmetic roughness parameter (*R_a_*). Table 4 shows the average and standard deviation of the arithmetic mean roughness values (*R_a_*). As expected, the surface treatments with larger sandpaper grain sizes (P40 and P80) implied higher surface roughness. Thus, *R_a_* clearly decreases as the number of particles of the sandpapers increases. 

### 3.2. Shear Testing

**Figure 4:**
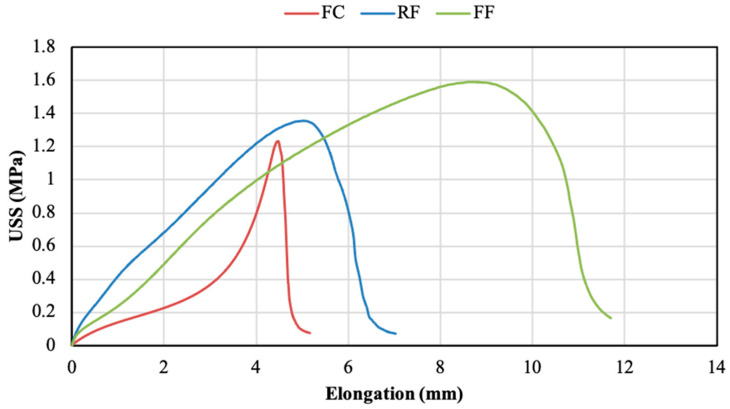
Characteristic shear stress–elongation curves of the 3 PSAs.

The typical behaviour of the three PSAs studied when subjected to shear stress is shown in Figure 4. The stress–strain curves represented in this figure correspond to joints using aluminium specimens ground with a sandpaper size of P1200 with the adhesion promoter. In these curves, a different shape is observed for each adhesive matrix (acrylic foam/acrylic core). Regarding the acrylic foam adhesives (RF and FF), the RF showed higher elastic modulus (stiffness) than the FF, whereas the FF presented a greater elastic deformation and strength. In these curves, the slope is slightly decreasing up to the maximum stress value, meaning that the stiffness slowly diminishes as these adhesives elongate. In the case of the acrylic core (FC), the curve exhibited a non-linear behaviour at the elastic zone, its slope gradually increasing up to the maximum strength. The different nature of the cores seems to be the reason for this stiffness change tendency. Concerning the strength, measured by the maximum shear stress values, the FF was the adhesive providing the highest values, whereas the FC was the one presenting the lowest resistance. The elongation of the adhesives prior to bond failure was also analysed. A higher elongation is directly related to a higher elasticity of the adhesive and, therefore, a better capacity to recover its original shape after the load release. According to the maximum ultimate shear strength (USS) and elongation, the FF adhesive provided the highest strength and ductility.

#### 3.2.1. Shear Results without Adhesion Promoter

**Figure 5:**
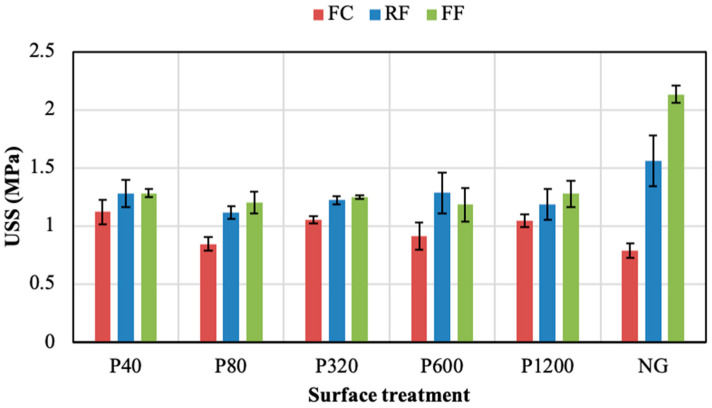
Ultimate shear strength (USS) of the adhesive joints under different surface treatments without adhesion promoter.

The influence of the grinding surface process on the PSA’s shear resistance was analysed as follows. Figure 5 reports the peak values (USS) obtained from the samples without the adhesion promoter. In the three PSAs, no clear relationship between USS and the grinding level was found in the ground samples. However, the PSAs displayed a different behaviour when the aluminium substrates were not ground (NG). Thus, the FC NG samples provided an USS value of 0.79 MPa. The values for the ground FC samples ranged between 0.84 and 1.12 MPa, with the peak (1.12 MPa) in the P40 condition. Regardless the surface treatment, FC always provided lower USS than RF and FF. Meanwhile, the joint shear resistance of both the RF and the FF notably increased when the NG condition was applied to the aluminium substrates. Concisely, the maximum values of the RF adhesive subjected to grinding processes ranged between 1.11 and 1.28 MPa, with 1.56 MPa for the NG condition. Similarly, the FF adhesive values ranged between 1.18 and 1.28 MPa for the ground samples, presenting the highest USS (2.13 MPa) when the substrates were not ground (NG). The FF was the PSA that provided the highest USS value among the three adhesives studied. 

**Figure 6:**
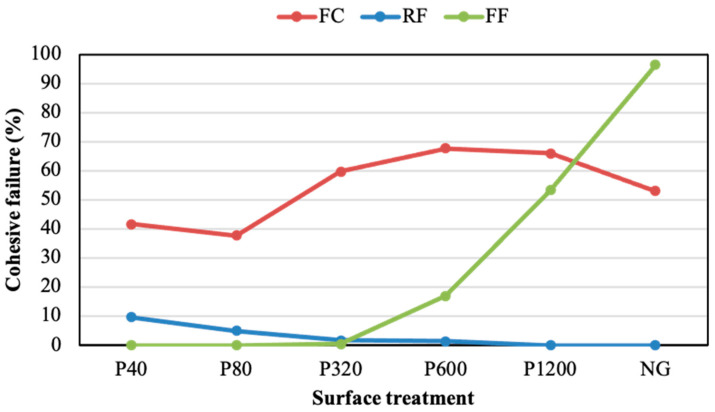
Cohesive failure percentage of adhesive joints under different surface treatments without adhesion promoter.

The fracture mode was studied in the samples, characterising the adhesive/cohesive rupture in terms of the percentage of cohesive failure. Figure 6 plots this percentage for joints subjected to different surface treatments without adhesion promoter. The percentage of cohesive failure of the joints assembled with the FF adhesive improved as the roughness reduced. This behaviour may be related to the decrease in the amount of air bubbles that were trapped in the interface, and consequently to the increase in the active surface of the joint. The strength of the joints assembled with the FC adhesive did not follow any clear trend as the surface roughness varied. The FC displayed the highest cohesive percentages in ground samples, although it showed the lowest shear resistance (Figure 6). This may be because the acrylic core adhesive (FC) exhibits higher formability than acrylic foam adhesives (RF and FF). This difference may lie in the fact that the FC adhesive has greater malleability than acrylic foam adhesives (RF and FF), presenting a better grip on rough substrates than on smooth substrates [44]. In addition, the cohesion of the FC joints was slightly favoured when the substrates had a roughness lower than 1 μm (obtained with sandpapers of P320, P600, P1200, and NG). The behaviour of this adhesive was similar to that described by Cui et al. [4], in which the adhesive based on epoxy and polyurethane did not present a linear trend between grain size sanding and USS. 

**Figure 7:**
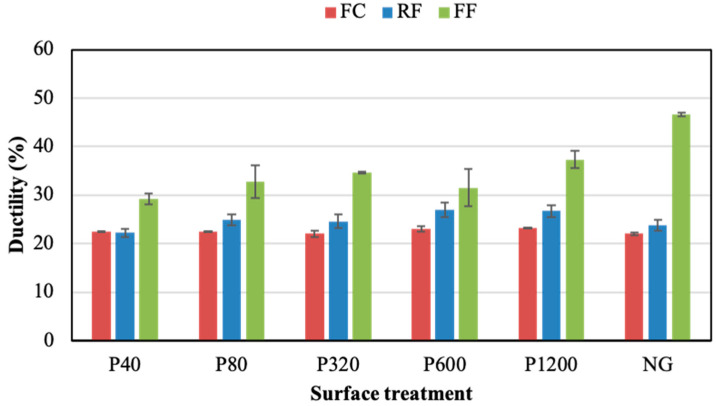
Ductility of adhesive joints under different surface treatments without adhesion promoter.

**Figure 8:**
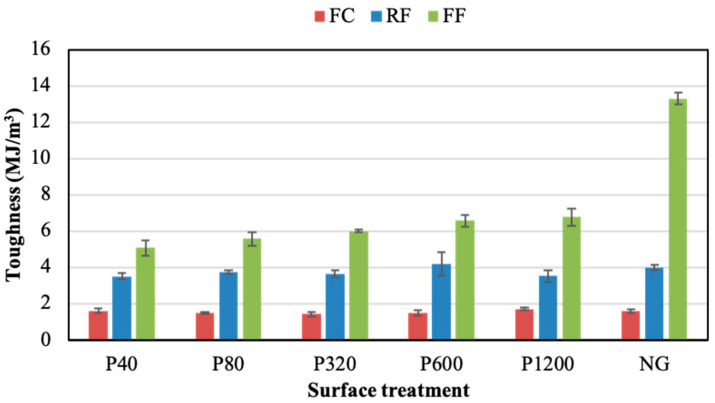
Toughness of adhesive joints under different surface treatments without adhesion promoter.

Figure 7 and Figure 8 show the ductility and toughness results of joints assembled without the adhesion promoter, respectively. Both properties did not seem to follow a clear trend when changing the grinding process. For both parameters, the FF adhesive showed higher values than the others, regardless of the grinding condition. The NG condition visibly led to the best ductility and toughness results for the three PSAs, especially in the case of the FF adhesive. Thus, the NG samples provided ductility values of 22.0%, 23.8%, and 46.6%, for the FC, RF, and FF, respectively. Meanwhile, the toughness of NG samples was 1.6 MJ/m^3^, 4.0 MJ/m^3^, and 13.3 MJ/m^3^ for the FC, RF, and FF, respectively.

Taking into account the overall results reported in this section, it is clear that FC adhesive present similar mechanical behaviour regardless the surface roughness. This is related its composition and production method, presenting a higher gelation than RF and FF adhesives. This property provides to FC a high flexibility and adaptability to the different roughness conditions tested, reporting similar USS, ductility, and toughness results. FC adhesive is known to contain trimethylolpropane triacrylate (TMPTA) with a high degree of crosslinking, providing the gelled texture [45]. 

Note that RF and FF adhesives do not have gel structures, having therefore less flexibility and adaptability to rough surfaces. Thus, RF and FF present a lower aluminium–adhesive contact area as the roughness is higher. This means that both adhesives show their best response when assembled on NG substrates. This behaviour is related to a high content of methyl methacrylate (MMA) in their compositions, providing a greater adhesion to substrates [45]. As the MMA polymer presents a lower crosslinking degree than TMPTA, the structure of the RF and FF adhesives does not present gel texture, reducing their adaptability to rough substrates, decreasing therefore the effective area of the union [45]. This explains why RF and FF (adhesives without gel texture) show their best mechanical behaviour in NG condition. 

In addition, according to the results obtained by other authors [46,47], an increase in the molecular weight of acrylic PSAs leads to a decrease in resistance. The density values reported in Table 2 show that FC presents a higher density than RF and FF, which is related to the higher molecular weight of TMPTA (C_15_H_20_O_6_) when compared to MMA (C_5_H_8_O_2_). The results obtained in the present study are in good agreement with previous investigations [46,47], as the highest molecular weight PSA (FC) leads to the lowest USS values. 

The deformability of crosslinked adhesives is also related to the molecular weight; an increase in molecular weight leads to an increase in PSA deformation. The recovery capacity increases with the cross-linking of the polymeric chains [48]. The crosslinking of the polymeric chains increases the flexibility and the elastic limit of the PSAs. As commented before, TMPTA has a more crosslinking degree and higher molecular weight than MMA. This is the reason why FC shows higher flexibility (lower slope at elastic region, observable in Figure 4) than RF and FF. The obtained results are therefore in good agreement with those reported by [48].

#### 3.2.2. Shear Results with Adhesion Promoter

**Figure 9:**
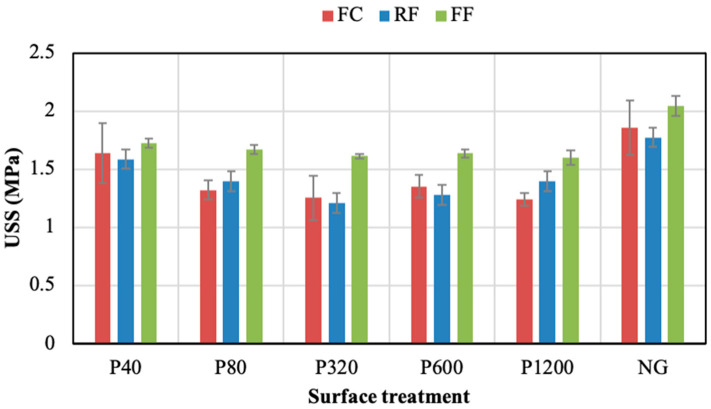
Ultimate shear strength (USS) of adhesive joints under different surface with adhesion promoter.

The shear performance developed by the adhesives when applying the adhesion promoter is presented in Figure 9. As observed, the application of this chemical treatment developed a higher mechanical performance of the three PSAs in most of the conditions. This behaviour was more pronounced when assembling aluminium substrates without sanding. 

**Figure 10:**
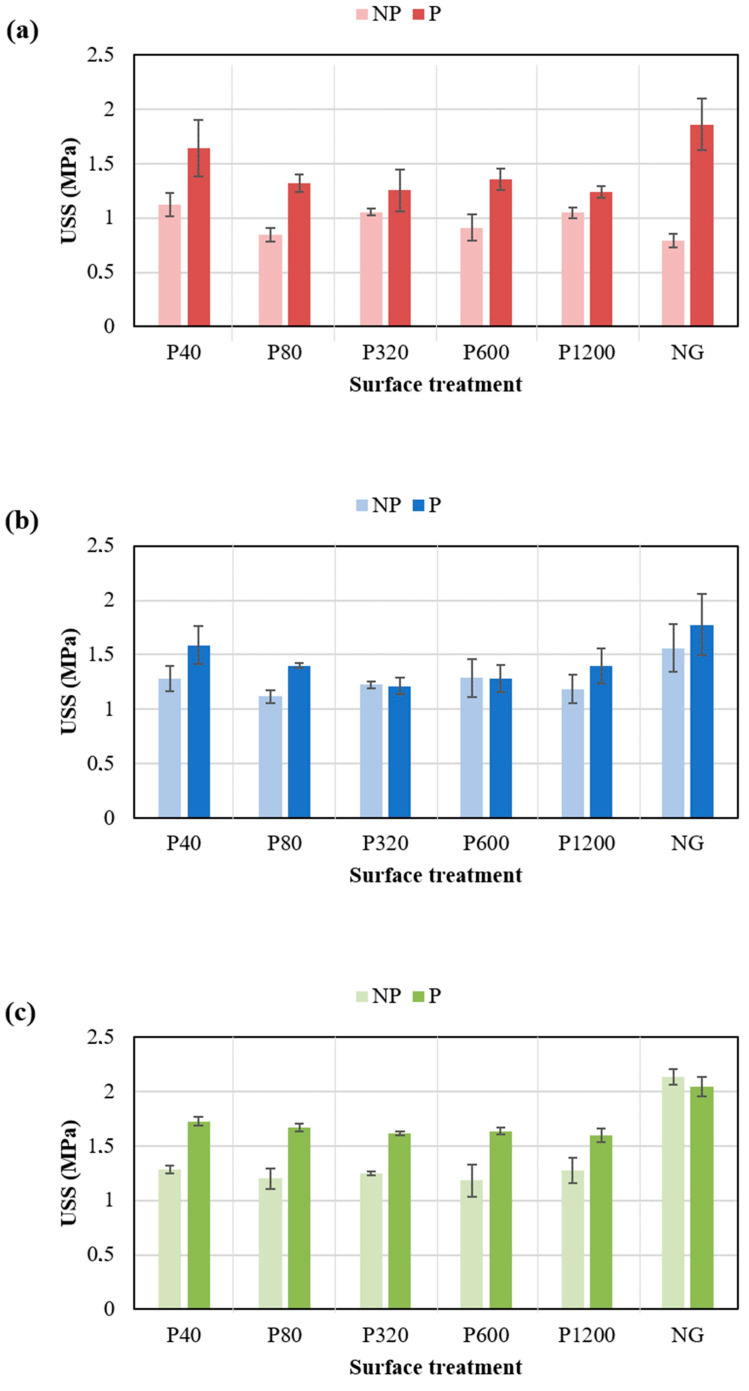
USS values of joints with (P) and without (NP) adhesion promoter for aluminium joints assembled with (**a**) FC, (**b**) RF, and (**c**) FF.

Generally, the joints manufactured with the FF adhesive displayed higher strength values compared with the other adhesives, independently of the condition used. For this adhesive, a significant increase in ultimate shear strength (USS) was observed after the application of the adhesion promoter, especially for ground substrates. The NG condition for this adhesive presented a similar behaviour with and without promoter. In the case of the RF adhesive, the use of the adhesion promoter also increased the USS. The highest result was observed for the NG condition. Regarding the FC adhesive, an important increase in USS was obtained with the promoter in all surface conditions, where the peak value was reached in the NG condition. Figure 10 compares the effect of using adhesion promoter in the USS values for the three PSAs studied. The figure clearly shows that the USS values of joints with promoter (P) are significantly higher than without promoter (NP) for the three PSAs.

Table 5 shows the improvement percentage (*IP*) data of shear resistance after the application of the adhesion promoter (*SR_P_*) with respect to the values obtained without the promoter (*SR_NP_*). These values were calculated for each adhesive and each grinding condition according to Equation (1):

(1) $$IP\;\left(\%\right)=\frac{SR_{P}-SR_{NP}}{SR_{NP}}*100$$

The results clearly demonstrate that the adhesion promoter enhanced the shear resistance of all adhesives. Note that the statistical error of these values is around 5%; therefore, the values below this percentage in this table are considered negligible. 

**Figure 11:**
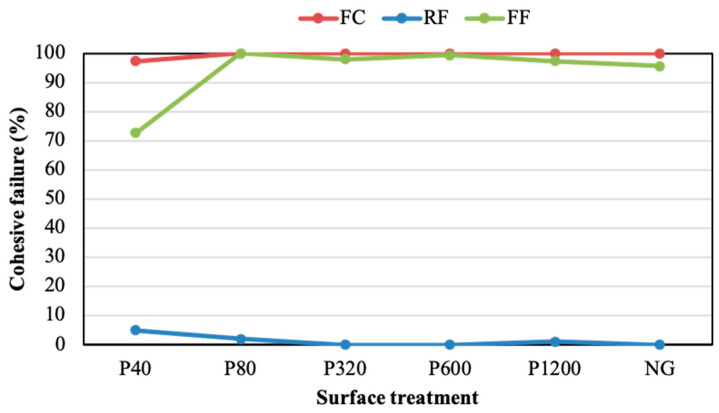
Cohesive failure percentage of the adhesive joints under different surface treatments with the adhesion promoter.

Figure 11 reports the cohesive failure percentage values obtained from the analysis of the fractured surfaces of adhesives with the adhesion promoter. It can be seen that the application of the promoter in sanded substrates supported a notable enhancement in the cohesion of the joints manufactured with the FF and the FC adhesives. This improvement was especially notable in the FC, where the use of the adhesion promoter developed a practically cohesive fracture (average of 99.55%) and only the P40 condition generated a percentage below 100%. These values indicate that the adhesive was properly adhered to the entire substrate area, effectively resisting to the shear forces imposed by the test. However, the RF adhesive hardly showed significant improvements in the cohesive failure percentage after the application of the promoter. The cohesion fracture of this adhesive displayed low percentages, similar to those reached without promoter, indicating that the adhesion between this PSA and the substrates was not appropriate, leading to poor mechanical resistance and adhesion fracture. In short, the FF and the FC developed cohesive fracture percentages close to 100%, with this value being always below 5% for the RF. The improvements observed for the FF and the FC adhesives were related to the chemical activation of the surface and the moldability capacity. Despite the application of the promoter, the RF adhesive did not seem to develop surface chemical activation. Its low moldability, related to its relatively high stiffness (Figure 4), may be the reason for this poor cohesion. 

**Figure 12:**
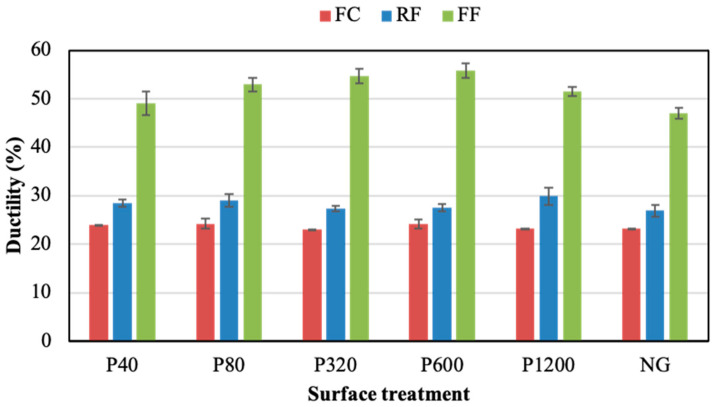
Ductility of the adhesive joints under different surface treatments with the adhesion promoter.

Figure 12 shows the ductility results of joints assembled with the adhesion promoter. Similar values were obtained for all grinding conditions. The promoter led to a clear ductility enhancement, especially for the FF adhesive. For the RF adhesive, the results obtained for the joints without the adhesion promoter reached an average of 24.8%, which increased to 28.2% when the adhesion promoter was applied. For joints assembled with the FF adhesive, an increase in ductility values from 35.4% to 52.8% was observed when the adhesion promoter was applied over the sanded substrates. For the FF joints assembled with NG condition, the ductility was similar with and without adhesion promoter (around 47%). In the case of the FC joints, the application of adhesion promoter did not improve significantly the ductility values (22–24%). 

**Figure 13:**
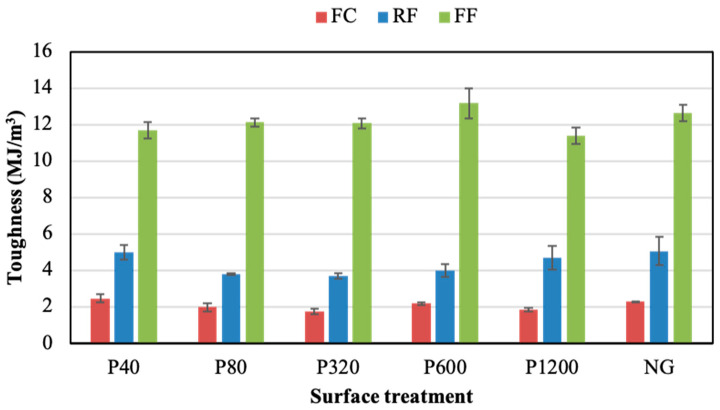
Toughness of the adhesive joints under different surface with the adhesion promoter.

The average toughness values for the three adhesive joints assembled using the adhesion promoter are shown in Figure 13. As observed, the values were not highly influenced by the surface preparation. Comparing the results of Figure 9 and Figure 13, it is clear that the use of the adhesion promoter improved the toughness of the three adhesives. On average, it increased from 3.8 to 4.4 MJ/m^3^ in the RF, from 1.6 MJ/m^3^ to 2.1 MJ/m^3^ in the FC joints, and from 6.0 MJ/m^3^ to 12.1 MJ/m^3^ in the ground FF joints. The most evident toughness increment was observed in the FF adhesive. For this PSA, the highest toughness was reported in the NG condition, regardless of the application of the adhesion promoter (NG condition without promoter provided relatively high toughness values).

The reported results demonstrate that the shear behaviour of the three PSAs was notably increased when the adhesion promoter was employed. The surface treatment did not lead to a high variation of mechanical behaviour when the adhesion promoter was applied, with the NG condition being generally better than ground surfaces. These outcomes are interesting for industrial applications, as the adhesion promoter involves an improvement in mechanical behaviour regardless of the surface roughness. This improvement was due to the increase in the Van der Waals bonds and electrostatic forces of the joints, as the use of the adhesion promoter notably improves the mechanical properties (USS, ductility and toughness) and delays the failure [49]. Additionally, it is also known that the adhesion promoter increases the surface energy of substrates [39].

Generally, acrylic adhesives have a weight composition of 3–10% pure acrylic acid and 90–97% alkyl acrylate [50,51]. The polymer crosslinking in the manufacture of PSA is determined by the chemical reaction between the crosslinking agent and the adhesive. Crosslinking process increases the yield strength and the resilience capacity of the adhesive, decreasing the deformation capacity. Likewise, an excessive degree of crosslinking leads to polymer gelation, which deteriorates the adhesive properties such as stickiness and ductility [52,53]. The acrylic core adhesive (FC) used in the present study presents a more gelled texture than foam acrylic adhesives (FF and RF). This may be due to the higher crosslinking processes of FC, which leads to lower deformability compared to the other two acrylic foam adhesives (Figure 4). The different structures of these acrylic backing adhesives seem to be the reason for the different adhesion patterns as the surface roughness is modified (Figure 5 and Figure 9). Thus, while the adhesion of FC is not affected by roughness, both FF and RF show a resistance decrease as the roughness increases (Figure 7 and Figure 12).

### 3.3. T-Peel Test

**Figure 14:**
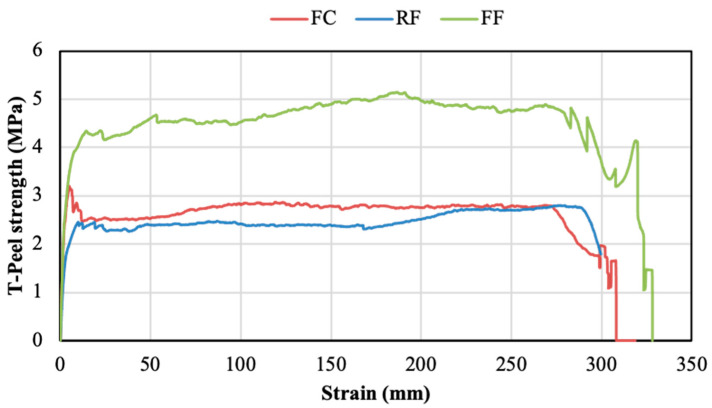
Characteristic T-peel stress–elongation curves of the 3 PSAs.

**Figure 15:**
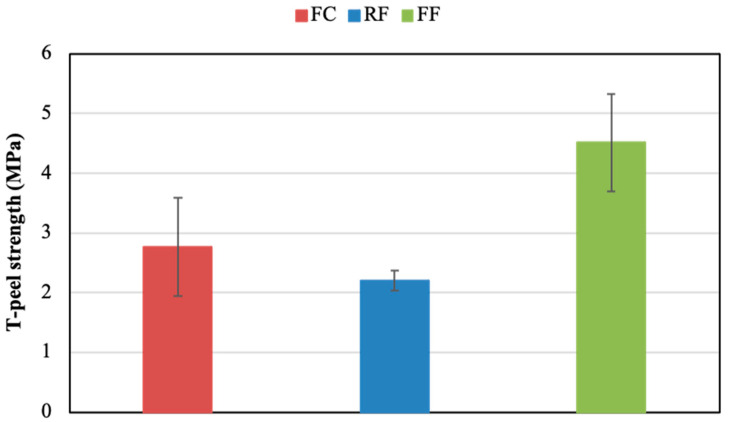
T-peel strength of the PSAs (NG condition with the adhesion promoter).

Taking into account that the NG condition was the easiest surface preparation and provided the best shear performance, this was the condition employed for the T-peel tests. Thus, T-peel tests were performed employing not sanded AA5754 H111 aluminium substrates of 0.5 mm thickness treated with the adhesion promoter. Representative T-peel stress–strain curves of the three adhesives are included in Figure 14 and the T-peel resistance (average of the plateau values of the curves) of all PSAs are represented in Figure 15. Similar to shear stress results, the highest T-peel strength was delivered by the FF adhesive, followed by the FC and the RF, with average values of 4.51 MPa, 2.76 MPa, and 2.20 MPa, respectively.

Table 6 reports the fracture mode expressed as the percentage of cohesive failure of adhesive joints subjected to T-peel tests. Both the RF and the FF displayed fully adhesive fractures. This fracture type changed for the FC joints, showing high T-peel cohesion. This disparity in the failure mode may be associated with the adhesive nature. Acrylic foam matrix adhesives (RF and FF) can present non-linear viscoelastic properties, assisted by the presence of bubbles in the foam. When these adhesives were subjected to stress, these bubbles started to deform, stretching and collapsing, causing a variation in the viscosity of the adhesive [54,55]. The different cohesion values for the shear and the T-peel tests were related to the direction of the adhesive matrix strain (note that shear and T-peel tests were performed at the same deformation rate). For the shear test, the strain direction was quasi-equatorial, while for the T-peel test the deformation occurred in the axial direction [56]. When the joints were subjected to shear, the movement occurred in a direction that did not allow the bubbles to grow, leading to a cohesive failure. In the T-peel tests, the strain was in the axial direction, permitting the bubbles’ growth. This led to a damping behaviour of the adhesive matrix, resulting in an adhesive failure. In the case of the FC adhesive, both the shear and the T-peel tests provoked cohesive behaviour, related to the homogenous matrix free of bubbles.

### 3.4. Creep Test

**Figure 16:**
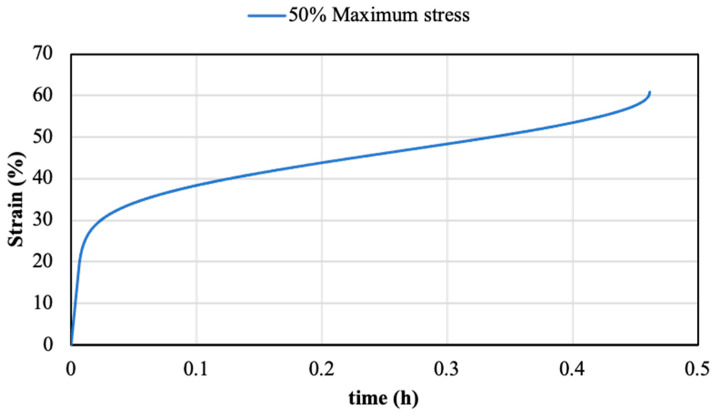
Characteristic creep curve of the FF under the 50% of the maximum shear tensile value.

The previous shear and T-peel tests reported the best mechanical behaviour for the FF adhesive, assembled over substrates that were not ground and treated with the adhesion promoter. Thus, the joints manufactured under this condition were selected to develop creep tests. After curing time, the joints were subjected to different loads. These loads were selected based on the maximum shear resistance (Section 3.2). The creep behaviour was characterised by the fracture time and the failure mode. These results are reported in Table 7. Figure 16 displays a typical creep behaviour curve obtained with a load of 20 kg/cm^2^ (50% of the maximum shear resistance). An inverse correlation between the failure time and the applied load was noted, with a significant lifetime increment with low loads. The adhesive demonstrated slight creep periods when loads of 1 to 10 Kg/cm^2^ were applied, indicating a maximum failure time of 115 h for the 1 Kg/cm^2^ load. To increase the creep fracture time, it is necessary to reduce the applied load to values below 0.5 Kg/cm^2^. This load value represents 2.5% of the maximum shear load. For the minimum load applied (0.25 Kg/cm^2^), the joints did not fracture after 8760 h (1 year). 

Regarding the fracture analysis, the maximum load supported by the FF adhesive (20 Kg/cm^2^) led to a total cohesive fracture. The results demonstrate that, as the load decreased, the type of fracture became more adhesive. This evolution in the adhesion properties was related to the critical stress required to initiate the cavitation process. Generally, cavity growth takes place as the joints are subjected to higher stress values, which provokes higher creep rates and shorter fracture times. This reduction in fracture time is due to a faster release of hydrostatic stress after the cavitation process [26,41,56,57,58,59]. Therefore, higher stress around the cavities promoted a reduction in the damping behaviour, increasing the cohesive fracture. 

**Figure 17:**
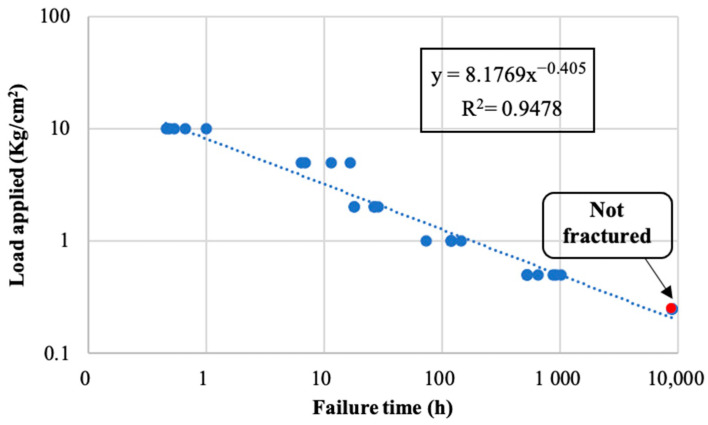
FF adhesive fracture time for the different loads applied.

Based on all data acquired for the FF joints subjected to the static loads, a simple modelling of the failure time was developed. Figure 17 plots the creep data, representing the load applied versus failure time in a double logarithmic scale. The linear equation better fitting by linear regression to these data is depicted in the figure. The high coefficient of determination value (R^2^) confirmed the high data fitting. Note that the result was an exponential equation type, provided by the double logarithmic scales. Using this model, the failure time of the FF adhesive joints can be estimated as a function of the applied load. Equation (2) depicts this fitting model.

(2) y=a+b·x

where *y* is log (applied load), *x* is the log (fracture time), and *a* and *b* are the fitting parameters, 0.913 Kg/cm^2^ and −0.405 Kg/(cm^2^·h), respectively.

## 4. Conclusions

The present work reported the mechanical behaviour of three acrylic pressure-sensitive adhesives (PSAs) assembled over aluminium specimens with different surface and chemical treatments. This study is in high demanded in some industries, such as the shipbuilding and offshore sectors, as the application of PSAs to join non-structural components to aluminium substrates has not been widely investigated. In this sense, shear, T-peel, and creep tests were performed, followed by surface fracture analysis. Based on these mechanical results, the following conclusions could be drawn:

- Low roughness values (with *R_a_* values equal to lower than 0.21 µm) notably improved the USS of the acrylic foam adhesives (RF and FF). Acrylic core adhesive (FC) presented similar behaviour regardless of the roughness.
- The application of adhesion promoter before assembling resulted in a significant improvement in USS and cohesion for the three adhesives.
- The FF adhesive displayed the best shear and T-peel behaviour.
- Creep tests performed with the FF adhesive allowed the estimation of a fitting equation relating the applied load and the failure time. Loads below 0.25 Kg/cm^2^ did not fracture after one year.

## Figures and Tables

**Table 1:** Chemical composition of the aluminium alloys (wt%).

Alloy	Mg	Mn	Fe	Si	Cr	Zn	Cu	Pb	Ti	Al
AA5083 H111	4.353	0.508	0.289	0.137	0.089	0.039	0.023	0.002	0.010	Bal.
AA5754 H111	2.783	0.232	0.322	0.154	0.044	0.043	0.037	0.022	0.019	Bal.

**Table 2:** Type and thickness of the adhesives.

Pressure-Sensitive Adhesive	Commercial Name	Thickness (μm)	Density (g/cm^3^)
Flexible Acrylic Core (FC)	7054, TESA, Germany	500	1.17 ± 0.08
Rigid Acrylic Foam (RF)	7044, TESA, Germany	1000	0.72 ± 0.07
Flexible Acrylic Foam (FF)	92111, TESA, Germany	1100	0.52 ± 0.04

**Table 3:** Different loads used in the creep tests.

Percentages of the Maximum Load Applied (%)	Load Value (kg/cm^2^)
50	10
25	5
10	2
5	1
2.5	0.5
1.25	0.25

**Table 4:** Aluminium roughness (average ± standard deviation of *R_a_* values) after different grinding preparations.

Surface Treatment	*R_a_* (μm)
P40	4.4 ± 0.7
P80	1.9 ± 0.3
P320	0.6 ± 0.1
P600	0.4 ± 0.2
P1200	0.3 ± 0.1
No Grinding (NG)	0.21 ± 0.02

**Table 5:** Improvement percentage (*IP*) data of shear joints resistance after the application of adhesion promoter (*SR_P_*) with respect to the values obtained without promoter (*SR_NP_*).

Surface Treatment/Adhesive	P40	P80	P320	P600	P1200	NG
FC (%)	46.3	56.3	18.9	48.3	18.6	136.1
RF (%)	24.1	25.2	0	0	18.0	13.8
FF (%)	34.4	39.1	29.0	38.2	25.4	0

**Table 6:** Percentage of cohesive failure of adhesive joints under the T-peel stress with the adhesion promoter.

**Cohesive** **Failure (%)**	**RF**	**FC**	**FF**
	0.0	79.5	0.1

**Table 7:** Average failure time and mode for different load values.

Load (kg/cm^2^)	Fracture Time (h)	Cohesive Failure (%)
10	0.63	100
5	10.39	10.96
2	23.55	4.91
1	115.40	1.51
0.5	692.00	1.50
0.25	>8760.00 (not fractured)	-

## Data Availability

Not applicable.

## References

[1] Ciardiello R. (2019). Mechanical characterization and separation tests of a thermoplastic reinforced adhesive used for automotive applications. Procedia Struct. Integr..

[2] Boutar Y., Naïmi S., Mezlini S., Carbas RJ C., da Silva LF M., Ben Sik Ali M. (2021). Cyclic fatigue testing: Assessment of polyurethane adhesive joints’ durability for bus structures’ aluminium assembly. J. Adv. Join. Process..

[3] Kawajiri K., Kobayashi M., Sakamoto K. (2020). Lightweight materials equal lightweight greenhouse gas emissions: A historical analysis of greenhouse gases of vehicle material substitution. J. Clean. Prod..

[4] Cui J., Wang S., Wang S., Chen S., Li G. (2020). Strength and failure analysis of adhesive single-lap joints under shear loading: Effects of surface morphologies and overlap zone parameters. J. Manuf. Process..

[5] Budhe S., Banea M.D., de Barros S., da Silva L.F.M. (2017). An updated review of adhesively bonded joints in composite materials. Int. J. Adhes. Adhes..

[6] Banea M.D., Rosioara M., Carbas RJ C., da Silva L.F.M. (2018). Multi-material adhesive joints for automotive industry. Compos. Part B Eng..

[7] Rudawska A. (2010). Adhesive joint strength of hybrid assemblies: Titanium sheet-composites and aluminium sheet-composites. Experimental and numerical verification. Int. J. Adhes. Adhes..

[8] Rudawska A., Worzakowska M., Bociąga E., Olewnik-Kruszkowska E. (2019). Investigation of selected properties of adhesive compositions based on epoxy resins. Int. J. Adhes. Adhes..

[9] Chang B., Shi Y., Dong S. (1999). Comparative studies on stresses in weld-bonded, spot-welded and adhesive-bonded joints. J. Mater. Process. Technol..

[10] Gierenz G., Karmann W., Gierenz G., Karmann W. (2001). Adhesive Tapes. Adhesives and Adhesive Tapes, 1st ed.

[11] Arenas J.M., Alía C., Narbón J.J., Ocaña R., González C. (2013). Considerations for the industrial application of structural adhesive joints in the aluminium-composite material bonding. Compos. Part B Eng..

[12] Kweon J.H., Jung J.W., Kim T.H., Choi J.H., Kim D.H. (2006). Failure of carbon composite-to-aluminum joints with combined mechanical fastening and adhesive bonding. Compos. Struct..

[13] Biel A., Alfredsson K.S., Carlberger T. (2014). Adhesive Tapes; Cohesive Laws for a Soft Layer. Procedia Mater. Sci..

[14] Antosik A.K., Bednarczyk P., Czech Z. (2018). Aging of silicone pressure-sensitive adhesives. Polym. Bull..

[15] Kostyuk A., Ignatenko V., Smirnova N., Brantseva T., Ilyin S., Antonov S. (2015). Rheology and adhesive properties of filled PIB-based pressure-sensitive adhesives. I. rheology and shear resistance. J. Adhes. Sci. Technol..

[16] Desroches G., Wang Y., Kubiak J., Macfarlane R. (2022). Crosslinking of pressure-sensitive adhesives with polymer-grafted nanoparticles. ACS Appl. Mater. Interfaces.

[17] Qie L., Dubé M.A. (2012). Influence of polymer microstructure on the performance of post-treated latex-based pressure sensitive adhesives. J. Appl. Polym. Sci..

[18] Shen H.Z., Zhang J.Y., Liu S.J., Liu G.D., Zhang L.Q., Qu X.W. (2008). Effect of the chain-transfer-agent content on the emulsion polymerization process and adhesive properties of poly (n-butyl acrylate-co-acrylic acid) latexes. J. Appl. Polym. Sci..

[19] Fang C., Jing Y., Zong Y., Lin Z. (2016). Effect of N,N-dimethylacrylamide (DMA) on the comprehensive properties of acrylic latex pressure sensitive adhesives. Int. J. Adhes. Adhes..

[20] Hayashida S., Sugaya T., Kuramoto S., Sato C. (2016). Impact strength of joints bonded with high-strength pressure-sensitive adhesive. Int. J. Adhes. Adhes..

[21] Horgnies M., Darque-Ceretti E., Felder E. (2007). Relationship between the fracture energy and the mechanical behaviour of pressure-sensitive adhesives. Int. J. Adhes. Adhes..

[22] Machado JJ M., Nunes PD P., Marques EA S., da Silva L.F.M. (2019). Adhesive joints using aluminium and CFRP substrates tested at low and high temperatures under quasi-static and impact conditions for the automotive industry. Compos. Part B Eng..

[23] Čolo A., Tasić P., Hajro I. (2020). Investigation of Primer Influence on Strength of Aluminium Specimens Bonded by VHB Tape. Lect. Notes. Netw. Syst..

[24] Rudawska A., Wahab M.A. (2021). Mechanical properties of adhesive joints made with pressure-sensitive adhesives. Stroj. Vestn./J. Mech. Eng..

[25] Ortega-Iguña M., Chludzinski M., Churiaque C., Dos Santos R.E., Porrúa-Lara M., Abad-Fraga F., Sánchez-Amaya J.M. (2021). Mechanical behaviour of double side high performance PSA adhesive applied to painted naval structures. Polym. Test..

[26] Gupta S.K., Shukla D.K. (2020). Effect of stress rate on shear strength of aluminium alloy single lap joints bonded with epoxy/nanoalumina adhesives. Int. J. Adhes. Adhes..

[27] Bartkowiak M., Czech Z., Mozelewska K., Kabatc J. (2020). Comparison between thermal crosslinkers based on melamine-formaldehyde and benzoguanamine resin and their influence on main performance of acrylic pressure-sensitive adhesives as tack, peel adhesion, shear strength and pot-life. Polym. Test..

[28] Roberge S., Dubé M. (2006). A The effect of particle size and composition on the performance of styrene/butyl acrylate miniemulsion-based PSAs. Polymer.

[29] Townsend B.W., Ohanehi D.C., Dillard D.A., Austin S.R., Salmon F., Gagnon D.R. (2011). Characterizing acrylic foam pressure sensitive adhesive tapes for structural glazing applications—Part I: DMA and ramp-to-fail results. Int. J. Adh. Adh..

[30] Townsend B.W., Ohanehi D.C., Dillard D.A., Austin S.R., Salmon F., Gagnon D.R. (2011). Characterizing acrylic foam pressure sensitive adhesive tapes for structural glazing applications—Part II: Creep rupture results. Int. J. Adhes. Adhes..

[31] Yamaguchi T., Morita H., Doi M. (2006). Modeling on debonding dynamics of pressure-sensitive adhesives. Eur. Phys. J. E.

[32] Gay C., Leibler L. (1999). Theory of Tackiness. Phys. Rev. Lett..

[33] Choong GY H., Endruweit A., De Focatiis D.S.A. (2021). Analysis of contact area in a continuous application-and-peel test method for prepreg tack. Int. J. Adhes. Adhes..

[34] Chikina I., Gay C. (2000). Cavitation in adhesives. Phys. Rev. Lett..

[35] Lakrout H., Sergot P., Creton C. (1999). Direct observation of cavitation and fibrillation in a probe tack experiment on model acrylic pressure-sensitive-adhesives. J. Adhes..

[36] Lindner A., Maevis T., Brummer R., Lühmann B., Creton C. (2004). Subcritical failure of soft acrylic adhesives under tensile stress. Langmuir.

[37] Sosson F., Chateauminois A., Creton C. (2005). Investigation of shear failure mechanisms of pressure-sensitive adhesives. J. Polym. Sci. Part B Polym. Phys..

[38] Varchanis S., Kordalis A., Dimakopoulos Y., Tsamopoulos J. (2021). Adhesion, cavitation, and fibrillation during the debonding process of pressure sensitive adhesives. Phys. Rev. Fluids..

[39] Feng C.W., Keong C.W., Hsueh Y.P., Wang Y.Y., Sue H.J. (2005). Modeling of long-term creep behavior of structural epoxy adhesives. Int. J. Adhes. Adhes..

[40] Park H.W., Seo H.S., Lee J.H., Shin S. (2020). Adhesion improvement of the acrylic pressure-sensitive adhesive to low-surface-energy substrates using silicone urethane dimethacrylates. Eur. Polym. J..

[41] Kalapat N., Amornsakchai T. (2012). Surface modification of biaxially oriented polypropylene (BOPP) film using acrylic acid-corona treatment: Part I. Properties and characterization of treated films. Surf. Coat. Technol..

[42] Kostyuk A.V., Ignatenko V.Y., Antonov S.V., Ilyin S.O. (2019). Effect of surface contamination on the durability and strength of stainless steel—Polyisobutylene pressure-sensitive adhesive bonds. Int. J. Adhes. Adhes..

[43] Mohammed I.K., Charalambides M.N., Kinloch A.J. (2015). Modelling the interfacial peeling of pressure-sensitive adhesives. J. Non-Newton. Fluid. Mech..

[44] Hanamertani A.S., Ahmed S. (2021). Probing the role of associative polymer on scCO_2_-foam strength and rheology enhancement in bulk and porous media for improving oil displacement efficiency. Energy.

[45] Jamaluddin J., Lee M.C. (2013). Properties of UV-curable solvent-free pressure sensitive adhesive. J. Adhes. Sci. Technol..

[46] Lee J., Lee T., Shim K., Park J., Kim H., Kim Y., Jung S. (2016). Molecular weight and crosslinking on the adhesion performance and flexibility of acrylic PSAs. J. Adhes. Sci. Technol..

[47] Kajtna J., Krajnc M. (2011). Solventless UV crosslinkable acrylic pressure sensitive adhesives. Int. J. Adhes. Adhes..

[48] Zosel A. (1998). The effect of fibrilation on the tack of pressure sensitive adhesives. Int. J. Adhes. Adhes..

[49] Daniloska V., Carretero P., Tomovska R., Asua J.M. (2014). High performance pressure sensitive adhesives by miniemulsion photopolymerization in a continuous tubular reactor. Polymer.

[50] Czech Z. (2003). Crosslinking of pressure sensitive adhesive based on water-borne acrylate. Polym. Int..

[51] Lee S.-W., Park J.-W., Park C.-H., Kwon Y.-E., Kim H.-J., Kim E.-A., Woo H.-S., Schwartz S., Rafailovich M., Sokolov J. (2013). Optical properties and UV-curing behaviors of optically clear PSA-TiO_2_ nano-composites. Int. J. Adhes. Adhes..

[52] Czech Z. (2006). Solvent-based pressure-sensitive adhesives for removable products. Int. J. Adhes. Adhes..

[53] Zhang X., Ding Y., Zhang G., Li L., Yan Y. (2011). Preparation and rheological studies on the solvent based acrylic pressure sensitive adhesives with different crosslinking density. Int. J. Adhes. Adhes..

[54] Huh C., Rossen W.R. (2008). Approximate Pore-Level Modeling for Apparent Viscosity of Polymer-Enhanced Foam in Porous Media. SPE J..

[55] Safouane M., Saint-Jalmes A., Bergeron V., Langevin D. (2006). Viscosity effects in foam drainage: Newtonian and non-Newtonian foaming fluids. Eur. Phys. J. E.

[56] Stigh U., Biel A. (2018). Effects of strain rate on the cohesive properties and fracture process of a pressure sensitive adhesive. Eng. Fract. Mech..

[57] Huang H., Dasgupta A., Singh N. (2021). Predictive mechanistic model of creep response of single-layered pressure-sensitive adhesive (PSA) joints. Materials.

[58] Liu L., Zhao H., Wang F., Xue P., Tian J. (2019). Rheological behavior and flow instability in capillary extrusion of ultrahigh-molecular-weight polyethylene/high-density polyethylene/nano-SiO_2_ blends. J. Appl. Polym. Sci..

[59] Huang H., Dasgupta A., Singh N. (2020). Predictive mechanistic model for single-layered pressure-sensitive adhesive (PSA) joints: Part I: Uniaxial tensile stress-strain response. Eur. Phys. J. E. Soft. Matter..

